# Network and 16S rRNA Sequencing-Combined Approach Provides Insightal Evidence of Vitamin K_2_ for Salt-Sensitive Hypertension

**DOI:** 10.3389/fnut.2021.639467

**Published:** 2021-02-24

**Authors:** Tian-hao Liu, Ming-hao Chen, Wan-qing Tu, Qiu-er Liang, Wen-cong Tao, Zhen Jin, Ya Xiao, Li-guo Chen

**Affiliations:** ^1^College of Chinese medicine, Jinan University, Guangzhou, China; ^2^College of medicine, Jinan University, Guangzhou, China

**Keywords:** network, gut bacteria, vitamin K_2_, salt-sensitive hypertension, mice

## Abstract

Vitamin K_2_ (VK2), found to act to treat hypertension, has been widely used in the food and pharmaceutical industries nowadays. However, the potential targets and molecular mechanisms of VK2 for salt-sensitive hypertension have not been fully investigated. Therefore, the study aimed to investigate the potential molecular mechanisms of VK2 for salt-sensitive hypertension using network pharmacology and 16S rRNA sequencing strategy. The network pharmacology-based findings from KEGG enrichment analysis revealed that VK2-treated salt-sensitive hypertension was mechanically associated with the complement and coagulation cascades, calcium signaling pathway, renin–angiotensin system, etc. A total of 29 different bacteria in an animal experiment after VK2 supplementation were screened and functionally enriched using PICRUSt2. Additionally, 10 signaling pathways were identified in which the renin–angiotensin system was found to be the potential molecular mechanisms with the greatest change in multiple and statistical significance. Moreover, the results of the renin–angiotensin system-related protein expression exhibited VK2-inhibited renin–angiotensin system in salt-induced hypertensive mice, which significantly verified the previous biological and functional prediction analysis. Finally, spearman correlation analysis showed the different bacteria such as *Dubosiella, Ileibacterium*, etc., had a positive or negative correlation with renin–angiotensin system-related proteins in salt-induced mice. In conclusion, the potential molecular mechanisms of VK2 for salt-sensitive hypertension may be beneficially achieved by the specific inhibition of the renin–angiotensin system, contributing to the development for a new preventive strategy of salt-sensitive hypertension.

## Introduction

Salt-sensitive hypertension refers to low renin type hypertension caused by relatively high salt intake for a long time (1). High salt intake can increase blood pressure and cause damage to target organs such as the heart, brain, and kidneys, while its pathological mechanism is that high salt intake leads to genetic sodium transport disorder, leading to abnormal sodium excretion and tendencies of kidney sodium retention (2). It is found that high-salt diet has a great impact on people's health, and about a larger number of people die of cardiovascular diseases in the world every year due to excessive salt intake (1). Even salt-sensitive hypertension has a high prevalence and serious harm, which has caused huge economic burden to society, families, and individuals (1, 2). Therefore, it is of great significance to study the effective drugs for the treatment of salt-sensitive hypertension.

Recent studies have confirmed that salt intake in human body is related to gut bacteria, which in turn affects blood pressure (3, 4). As the most complex micro ecosystem of the human body, gut bacteria affect human health from many aspects, such as digestion, nutrition absorption, energy supply, fat metabolism, immune regulation, drug metabolism, and toxicity (5, 6). It was found that dietary sodium reduction increases circulating short-chain fatty acids, which are associated with decreased blood pressures, supporting that dietary sodium may influence the gut microbiome (4). In addition to its effect on blood pressure, salt can even act as an independent risk factor for target organ damage (3). Studies have shown that after 4 weeks of high-salt diet, the composition and function of fecal bacteria in mice were changed, resulting in imbalance in the proportion of regulatory T cells and pro-inflammatory helper T cells (Treg/Th17) (3).

Vitamin K_2_ (VK2) is a fat-soluble vitamin, mainly produced by bacterial synthesis. Studies have shown that VK2 mainly acts on extrahepatic tissues such as bone, brain, blood vessels, pancreas, kidneys, and lungs to activate K-dependent proteins such as osteocalcin and matrix Gla protein. It has become a focus of research in recent years and has been widely used in the food and pharmaceutical industries. Bentley et al. (7) briefly illustrated VK2 biosynthesis pathway related to bacteria in 1971. Ponziani et al. (8) found that gut bacteria was the main source of VK2 in humans and small intestinal bacterial overgrowth (SIBO) was associated with altered VK2 metabolism. Moreover, a higher intake of vitamin K_2_ produced by gut bacteria was associated with lower risk of coronary heart disease (CHD) (9). A meta-analysis showed that VK2 supplementation might prove to be of benefit as a long-term strategy to improve vascular health and reduce cardiovascular risk (9). In addition, Vissers et al. (10) found that a high intake of VK2 was significantly associated with a reduced risk of peripheral arterial disease, including hypertensive participants. It has been shown that the synergistic effect of VK2 and angiotensin-converting enzyme inhibitor (11, 12). All in all, it has been reported that VK2 is related to cardiovascular diseases, even hypertension. However, the molecular mechanism of its pharmacology has not been fully investigated.

Network pharmacology is based on the principle of system biology to explain the process of disease, and further use the holistic view of network structure to understand the mechanisms of drug and disease. In recent years, as a hot method, it has been widely used in the analysis of drug mechanism (13, 14). From the perspective of network pharmacology, the potential targets and mechanisms of VK2 on salt-sensitive hypertension were studied by using various database resources. Then the mice were supplemented with VK2 through animal experiment, the gut bacteria of mice was detected by 16S rRNA and functionally enriched using PICRUSt2. Finally, the common signaling pathway-related proteins in mice were detected to further verify the signaling pathways before the validation of clinical samples.

## Methods

### Screening Targets of Vitamin K_2_-Treated Salt-Sensitive Hypertension

The structure file of VK2 was downloaded in the PubChem database (https://pubchem.ncbi.nlm.nih.gov, searched on June 22, 2020) by searching for the keyword as “vitamin K_2_.” The targets of VK2 were obtained by SwissTargetPrediction (http://www.swisstargetprediction.ch, searched on June 22, 2020) and DRAR-CPI (https://cpi.bio-x.cn/drar/, searched on June 22, 2020) using the structure file of VK2. Then the target library of VK2 was constructed using WPS Office software 2019. Salt-sensitive hypertension related therapeutic targets were obtained using the GeneCards database (https://www.genecards.org, searched on June 22, 2020) and Omim database (https://omim.org, searched on June 22, 2020). The “salt-sensitive hypertension” was used to act as searching keyword. Then, the names of target proteins were transformed into the corresponding gene symbols by the UniProt database (https://www.uniprot.org, searched on June 23, 2020).

### Screening of Targets of Vitamin K_2_-Treated Salt-Sensitive Hypertension and the Construction of the Interrelated Network

The common targets library between putative targets of VK2 and the known therapeutic targets on salt-sensitive hypertension were amalgamated. Then the PPI network was integrated and conducted using the String database (https://string-db.org) according to the common targets.

### Analysis of Functional Processes and Molecular Pathways of Vitamin K_2_-Treated Salt-Sensitive Hypertension

The KEGG pathway enrichment analysis of common targets was carried out using clusterProfiler package which was used as a software package for pathway enrichment analysis and visualization in the R language (version 3.6.1). The screening condition was set as *p* < 0.001.

### Network Construction of Vitamin K_2_-Targets-Pathways-Disease Network

The network construction of VK2-targets-pathways-disease network was conducted using Cytoscape_v3.7.1. The detailed methods were described in previous reports (13, 14).

### Animals and Experimental Protocols

Eighteen 8-week-old male C57BL/6J mice were purchased from the experimental animal center of Guangzhou University of traditional Chinese medicine and raised in the animal center of Jinan University. All mice were naturally reared in a barrier environment with 12/12 h light cycle, 20–24°C and 40–60% humidity. This animal experiment was approved by the experimental animal ethics committee of Jinan University, which conforms to the principles of animal protection, animal welfare, and ethics, and the relevant provisions of national experimental animal welfare ethics. All mice were randomly divided into three groups: normal group (ND), high salt model group (HS), high salt diet plus VK2 supplementation group (HS_VK2), six mice per group. ND group was fed with a natural diet (containing 0.5% NaCl); HS group was fed with a high salt diet (containing 8% NaCl); HS_VK2 group was fed with high salt diet (containing 8% NaCl and additional 0.025% VK2) for 4 weeks. Finally, the mice were injected with 3% pentobarbital sodium to avoid suffering pain. After the necks were removed and sacrificed, the colon contents of the mice were quickly collected in sterile 1.5-ml EP tubes and stored at −80°C for testing.

### Monitoring of Blood Pressure

The systolic blood pressure of all mice before and after the experiment was monitored by tail artery manometry and took the average value using the blood pressure analysis program (BP2000, USA) according to the operation instructions. The temperature of the mouse platform was set to 37°C in advance and test 15 times in each round.

### Transmission Electronic Microscope Examination

The mouse aortic tissue was fixed in 2.5% glutaraldehyde at 4°C for 2–4 h under the condition of minimizing the mechanical injury such as traction, contusion, and extrusion. Then, the mouse aortic tissue was fixed and rinsed three times, 15 min each time, using 0.1 M phosphate buffer Pb (pH7.4). After dehydration and infiltration, the aortic tissue of mice was cut into 60–80 nm ultrathin sections. These sections were stained with uranium and lead (2% uranium acetate saturated alcohol solution, lead citrate, each staining for 15 min), and further observed under the TEM (HT7700; Hitachi; Tokyo, Japan), while three images were collected and analyzed from each group.

### Microbial Analysis

#### DNA Extraction and PCR Amplification

Four mice were randomly selected from each group and DNA was extracted from fecal samples using the E.Z.N.A.® soil DNA Kit (Omega Bio-tek, Norcross, GA, U.S.) according to the manufacturer's protocols. The final DNA concentration and purification were determined by NanoDrop 2000 UV-vis spectrophotometer (Thermo Scientific, Wilmington, USA), and DNA quality was checked by 1% agarose gel electrophoresis. The V3–V4 hypervariable regions of the bacteria 16S rRNA gene were amplified with primers 338F (5′-ACTCCTACGGGAGGCAGCAG-3′) and 806R (5′-GGACTACHVGGGTWTCTAAT-3′) by thermocycler PCR system (GeneAmp 9700, ABI, USA). The resulted PCR products were extracted from a 2% agarose gel and further purified using the AxyPrep DNA Gel Extraction Kit (Axygen Biosciences, Union City, CA, USA) and quantified using QuantiFluor™-ST (Promega, USA) according to the manufacturer's protocol. The detailed methods were described in previous reports (14).

#### Illumina MiSeq Sequencing

Purified amplicons were pooled in equimolar and paired-end sequenced (2 × 300) on an Illumina MiSeq platform (Illumina, San Diego, USA) according to the standard protocols by Majorbio Bio-Pharm Technology Co., Ltd. (Shanghai, China).

#### Processing of Sequencing Data

Operational taxonomic units (OTUs) were clustered with 97% similarity using UPARSE (version 7.1 http://drive5.com/uparse/) and each 16S rRNA gene sequence was analyzed using a confidence threshold of 70% in RDP Classifier algorithm (http://rdp.cme.msu.edu/) against the Silva (SSU123) 16S rRNA database. PICRUSt2 was used to predict microbial functions according to the normalized OTU tables (15).

### Measurements of Renin–Angiotensin System-Related Protein Expression by Western Blotting

Three mice were randomly selected from each group and the proteins in their aortic tissues were detected through Western blotting. Ren antibody (abcam, ab212197), ACE (abcam, ab254222), AT1R (abcam, ab124734), and AT2R (abcam, ab227851) antibodies were used in the current study. According to the kit instructions, the total proteins were obtained by conventional tissue. Thus, PAGE separation was conducted after equivalent sampling. PAGE separins used 10% separating and 5% stacking gel. All proteins separated by PAGE were transferred to PVDF membranes with primary and secondary antibodies added. Moreover, the proteins were exposed, developed, and fixed. GAPDH was used as an internal control. Quantity One 4.0 software was used to analyze the imaging map and the ratios of Ren, ACE, AT1R, and AT2R proteins in each group were calculated.

### Statistical Analysis

Statistical analysis on the gut microbiota was performed using R package (MathSoft, Inc., United States). All other data are presented as mean ± standard error of mean (SEM). Statistical analysis was performed using Student *t*-test or one-way analysis of variance (ANOVA) through GraphPad Prism 5 (San Diego, CA, USA). *p* < 0.05 was considered as statistically significant.

## Results

### Screening Target Information and PPI Network of Vitamin K_2_ on Salt-Sensitive Hypertension

**Figure 1:**
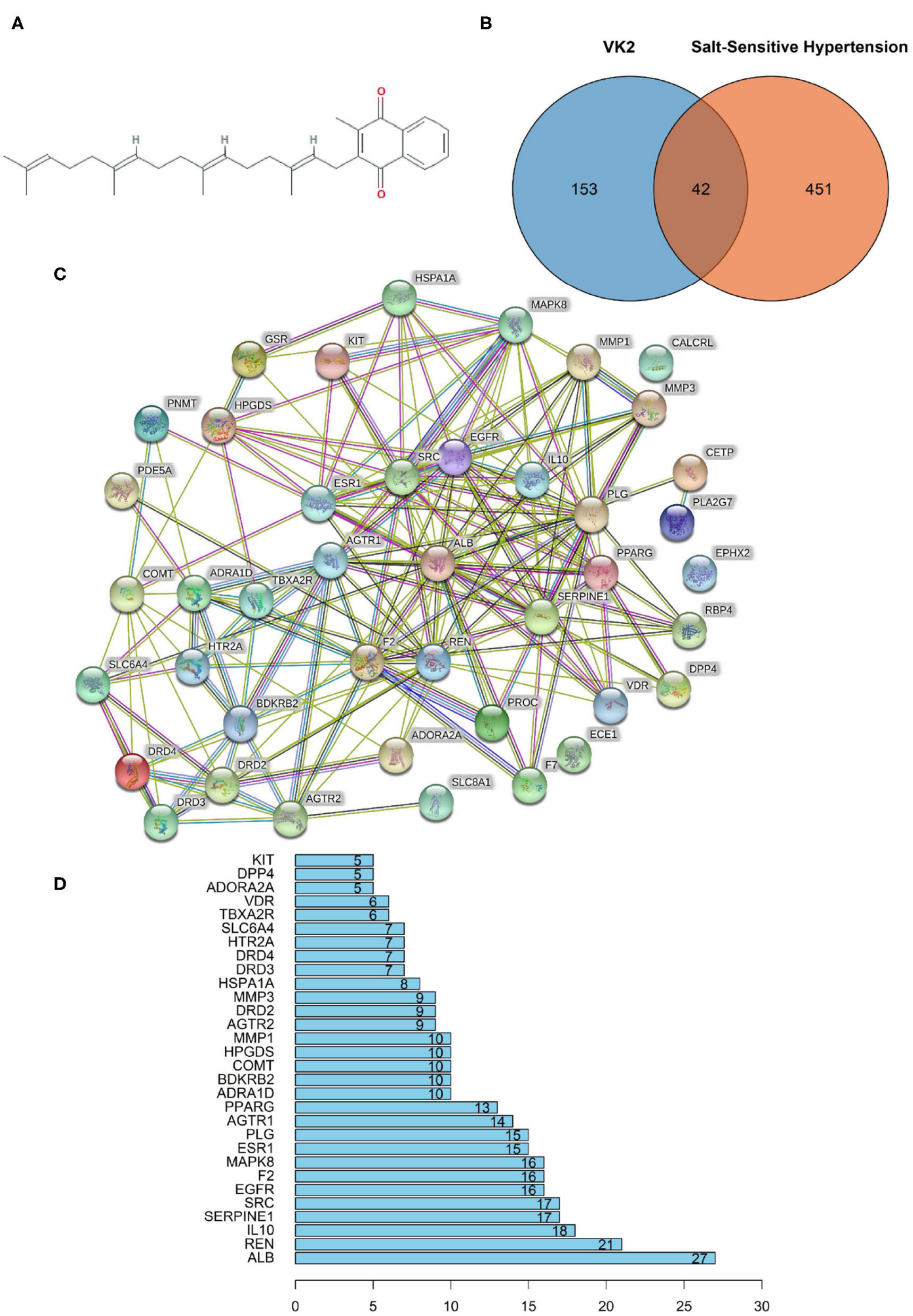
Network pharmacology revealed the target characteristics of Vitamin K_2_ (VK2) for salt-sensitive hypertension. **(A)** Molecule formula of VK2. **(B)** Venn diagram of common targets of VK2 for salt-sensitive hypertension. **(C)** The protein-protein interaction (PPI) network based on targets of VK2 for salt-sensitive hypertension. Nodes represent different proteins. Edges represent protein-protein associations, the line thickness indicates the strength of data support. **(D)** The top 10 core genes of VK2 for salt-sensitive hypertension.

The molecular formula of VK2 was C_31_H_40_O_2_, and the molecule structure was shown as Figure 1A. A total of 195 reported pharmacological targets related to VK2 were obtained from the databases (Supplementary Table 1). A total of 493 salt-sensitive hypertension related targets were obtained from the databases (Supplementary Table 2). Combining the common targets of VK2 and salt-sensitive hypertension, 42 targets were screened to be the targets of VK2-treated salt-sensitive hypertension (Figure 1B; Supplementary Table 3). The function-related PPI network was conducted using the STRING database and shown in Figure 1C. The network containing 42 nodes and 185 edges, had significantly more interactions (*p* < 1.0 × e^−16^). Additionally, the top 10 core genes were screened as ALB, REN, IL10, SERPINE1, SRC, EGFR, F2, MAPK8, ESR1, and PLG (Figure 1D; Supplementary Table 4).

### KEGG Pathway Enrichment of Core Targets and Construction of an Integrative Network

**Figure 2:**
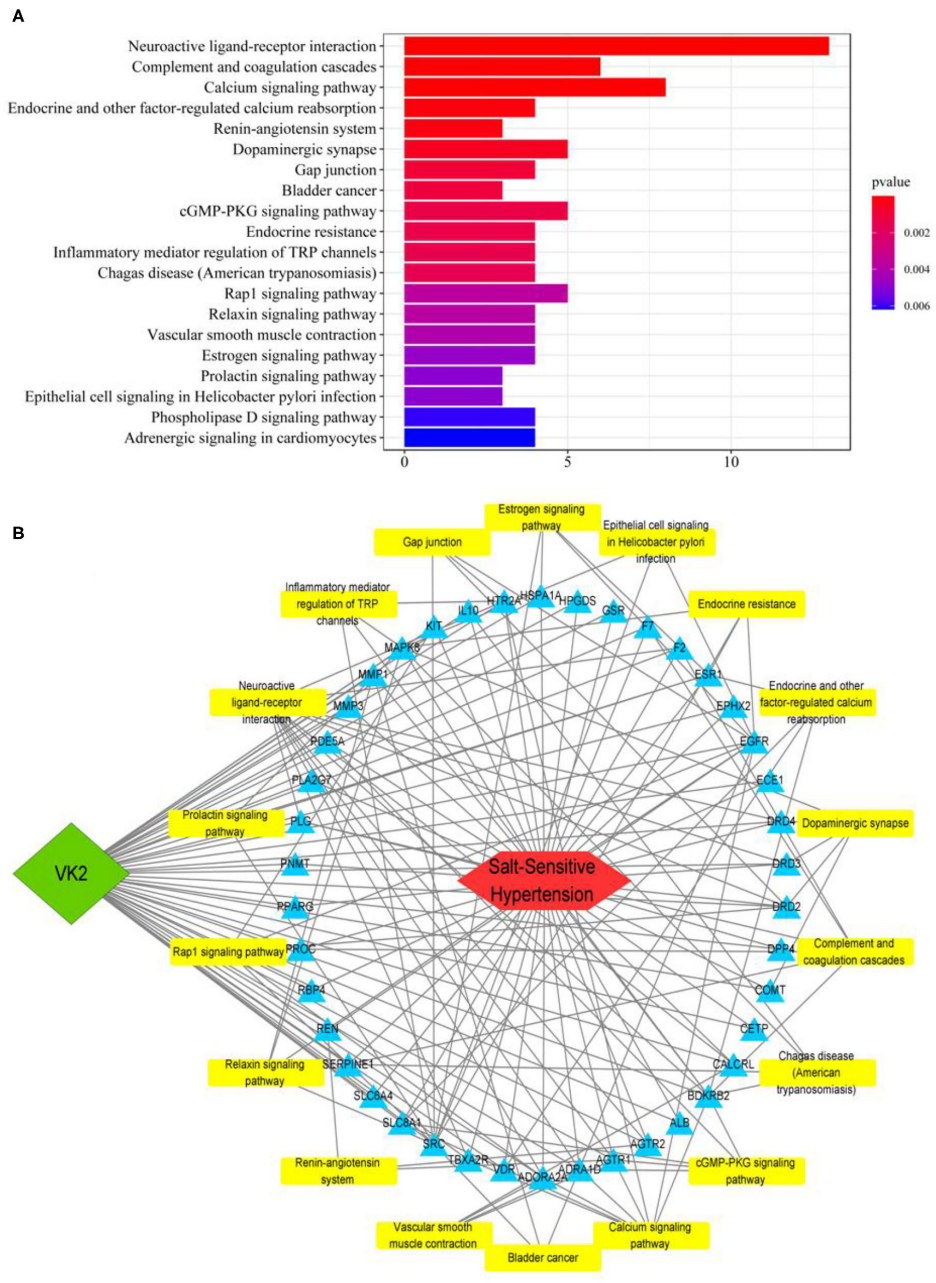
KEGG pathway enrichment of core targets and construction of an integrative network. **(A)** The bar diagram of KEGG pathway enrichment. **(B)** The integrative network of VK2 for salt-sensitive hypertension through the network pharmacology-based findings.

The KEGG pathway enrichment involved in predicted targets is systematically elucidated based on the KEGG pathway database. In the current study, KEGG pathway enrichment was performed according to the common 42 genes, and the top 20 enrichment data were plotted as a bar diagram (Figure 2A; Supplementary Table 5). The KEGG pathway enrichment mainly involved in neuroactive ligand-receptor interaction, complement and coagulation cascades, calcium signaling pathway, endocrine, and other factor-regulated calcium reabsorption, and renin–angiotensin system, etc. The integrative network of VK2 for salt-sensitive hypertension through the network pharmacology-based findings was conducted and shown in Figure 2B.

### Vitamin K_2_ Supplementation Protected Blood Pressure and Aortic Vessels in High Salt-Induced Mice

**Figure 3:**
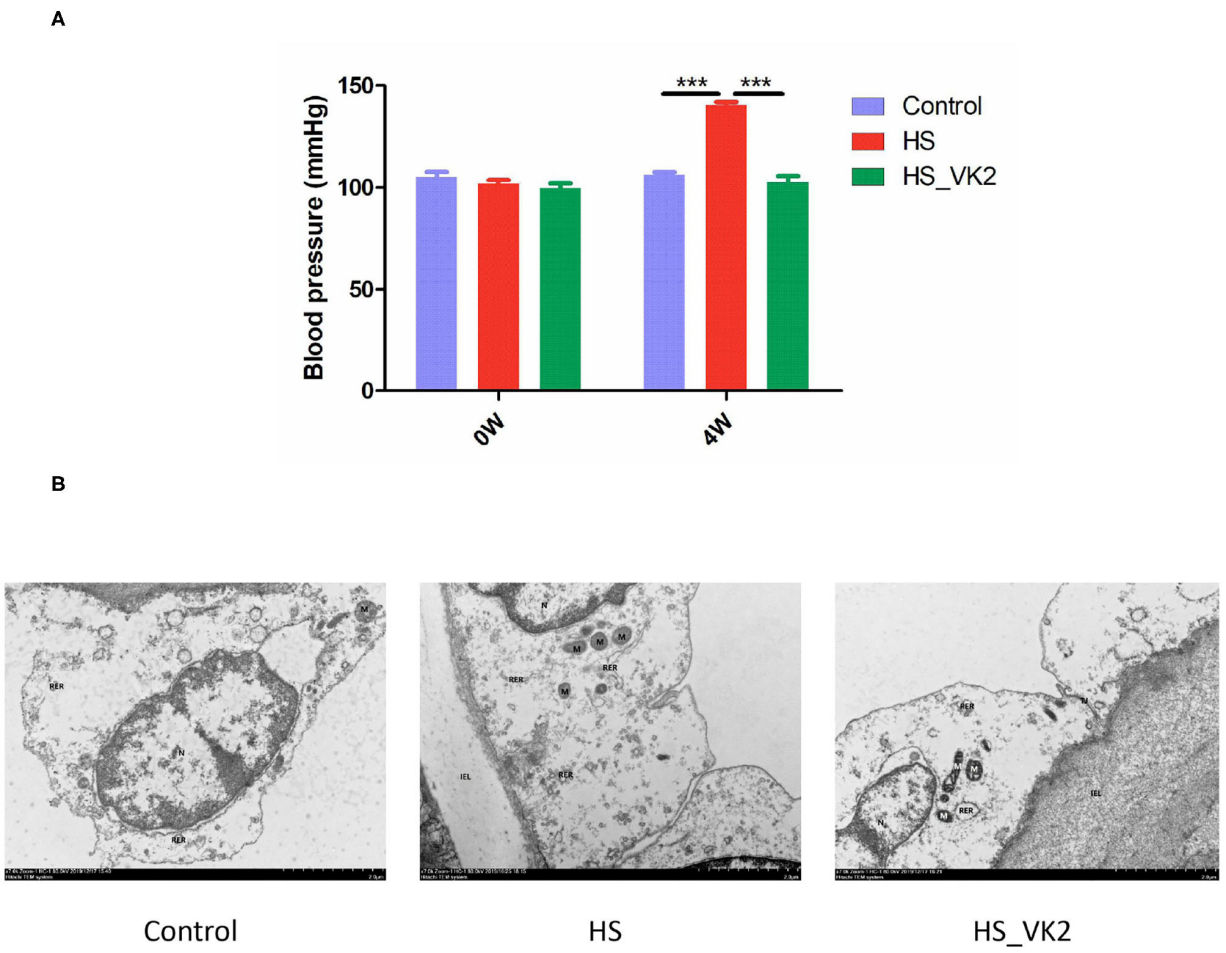
VK2 supplementation protected blood pressure and aortic vessels in salt-induced mice. **(A)** Blood pressure (mmHg). **(B)** The ultrastructural changes of aortic vessels observed by Transmission Electronic Microscope (TEM). Internal elastic lamina (IEL), nucleus (N), mitochondrion (M), rough endoplasmic reticulum (RER), tight junction (TJ), autophagy (AP). Data are presented as mean ± standard error of mean (SEM). ***p < 0.001, *n* = 6; statistical comparisons were performed using Student *t*-test or one-way analysis of variance (ANOVA).

Vascular endothelial cells maintain the normal flow of blood and act as a barrier between blood and tissue fluid, whose injury is related to the occurrence of hypertension (16, 17). Therefore, the blood pressure and the ultrastructural changes of aortic vessels were observed in animal experiment. The results of animal experiment showed that significantly increased blood pressure was found in the HS group compared with the control group, whereas a significantly decreased blood pressure was found after VK2 supplementation compared with the HS group (Figure 3A). Moreover, the results of ultrastructural changes of aortic vessels are shown in Figure 3B. Compared with the control group, the vascular endothelial cells showed obvious edema, the intracellular matrix became lighter, the electron density of large area decreased, the internal elastic membrane appeared obvious local fracture; the nucleus showed irregular shape, local depression, heterochromatin edge set; the number of mitochondria decreased, swelling, mitochondrial cristae became shorter, and disappeared; and the rough endoplasmic reticulum showed obvious expansion and degranulation (Figure 3B). After VK2 supplementation, the results showed that the edema of vascular endothelial cells was alleviated; the electron density of intracellular matrix was low and the transparency was reduced; the internal elastic membrane was not obviously broken; the damage of cell structure such as nucleus, mitochondria, and rough endoplasmic reticulum was alleviated (Figure 3B).

### General Bacterial Structural Characteristics After Vitamin K_2_ Supplementation in High Salt-Induced Mice

**Figure 4:**
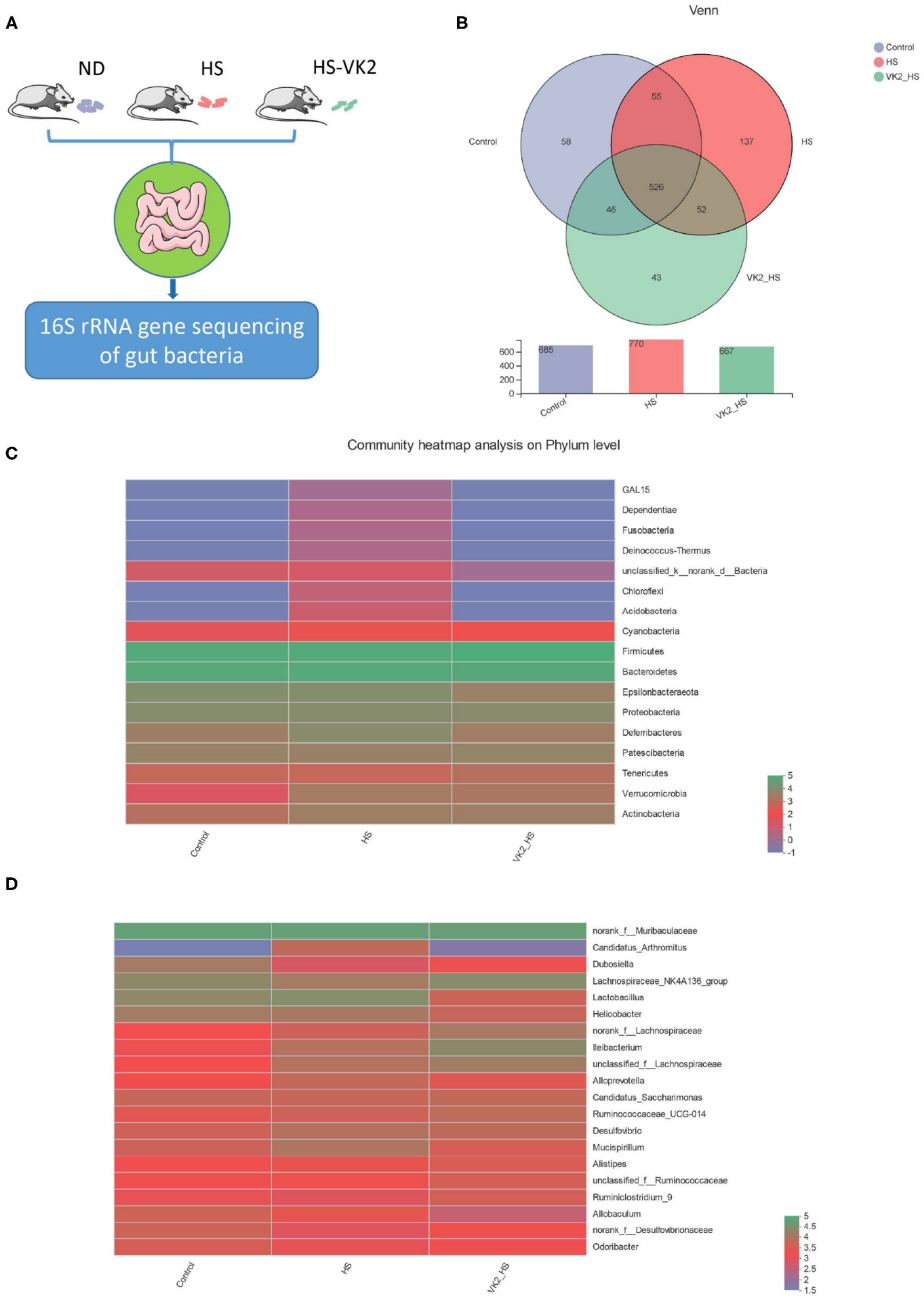
General bacterial structural characteristics after VK2 supplementation (*n* = 4). **(A)** The process of animal experimental verification. **(B)** The operational taxonomic units (OTUs) changes after VK2 supplementation. **(C)** Community heatmap analysis on phylum level after VK2 supplementation. **(D)** Community heatmap analysis on genus level after VK2 supplementation. Color represents relative abundance.

In order to further verify the potential mechanism of VK2 resistance to salt-sensitive hypertension, we supplemented VK2 to intervene in high-salt induced mice, and detected the characteristics of gut bacteria by 16S rRNA analysis (Figure 4A) The results showed that there were 690 total OTUs in the control group, 771 total OTUs in the HS group, 663 total OTUs in VK2_HS group, and 530 common OTUs in the three groups (Figure 4B). Among them, there were 585 common OTUs in the control group and HS group, 59 personalized OTUs in the control group and 138 personalized OTUs in the HS group, which indicated that OTUs of high salt diet were higher than those of normal diet. There were 667 common OTUs in the HS group and VK2_ HS group, 39 personalized OTUs in the VK2_HS group, 139 personalized OTUs in the HS group, which indicated that VK2 supplementation reduced OTUs in the gut bacteria of mice fed with high salt diet.

Furthermore, phyla level results showed that the proportions of dominant phyla Firmicutes, Bacteroidetes, and Proteobacteria in the control group were 41.26, 40.06, and 5.823%, whereas 43.32, 36, and 4.899% were in the HS group, and 49.99, 34.89, and 4.665% were in the VK2-HS group, respectively, (Figure 4C; Supplementary Table 6). In addition, 20 dominant genera were found in bacteria (Figure 4D; Supplementary Table 7). For instance, the relative abundances of *norank_f__Muribaculaceae, Lactobacillus, Lachnospiraceae_NK4A136_group, Helicobacter* in the control group were 34.49, 11, 10.54%, and 6.87; 29.07, 15.60, 5.83, and 5.24% were in the HS group; and 27.29, 2.17, 12.16, and 2.33% were in the VK2_HS group. All these findings revealed that there were differences in bacterial composition and structure after VK2 supplementation.

### Screening of Different Bacteria After Vitamin K_2_ Supplementation in High Salt-Induced Mice

**Figure 5:**
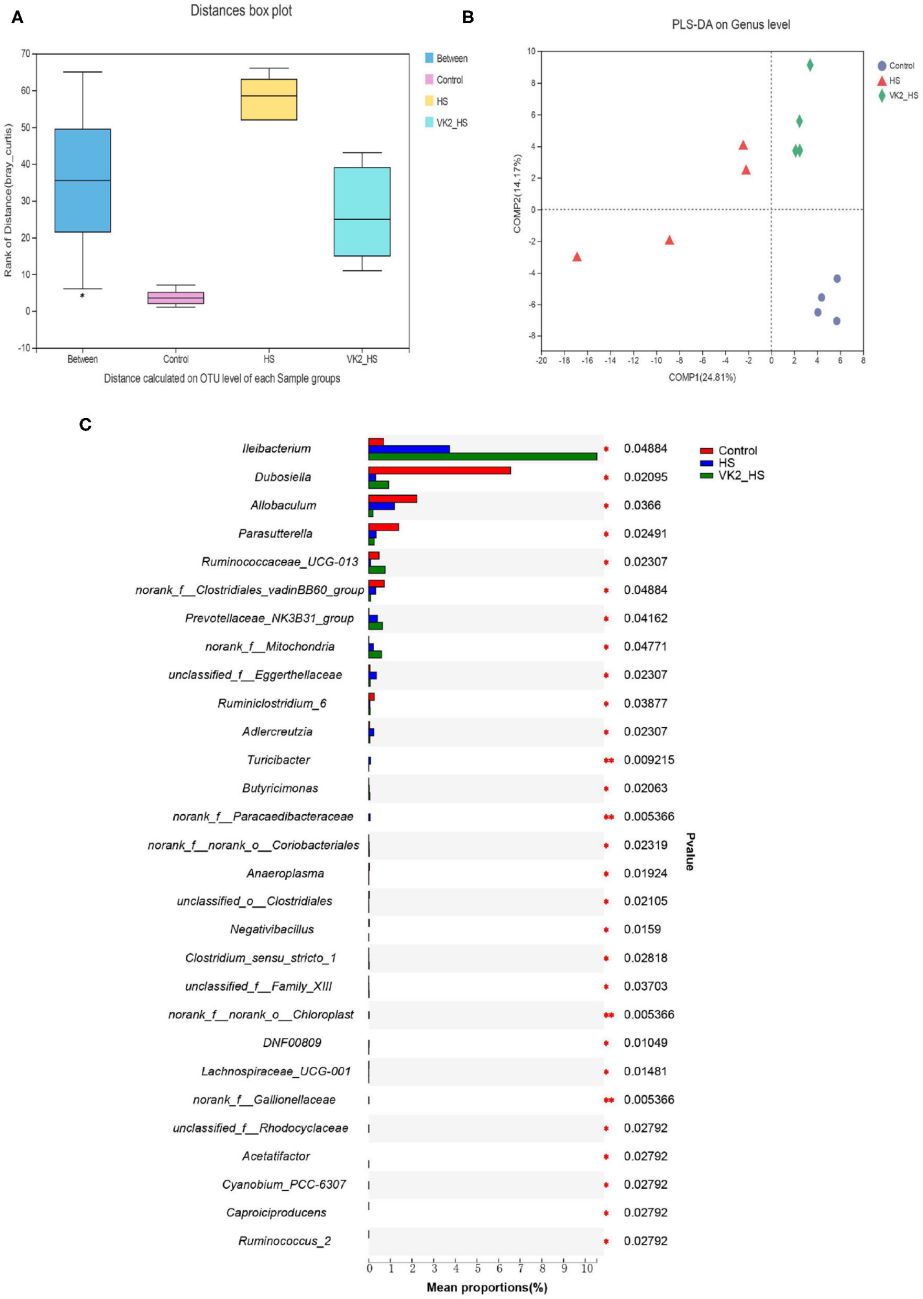
Screening of different bacteria after VK2 supplementation. **(A)** Distance calculated on OTU level. **(B)** PLS-DA. **(C)** Differential bacteria on genus level. *p < 0.05 and **p < 0.01, *n* = 4. Statistical comparisons were performed using one-way analysis of variance (ANOVA).

Similarities of the bacterial composition and structure after VK2 supplementation among groups were compared using ANOSIM and PLS-DA based on Bray–Curtiss (18). The results distance box plot showed that there were significant differences in the bacterial composition and structure (Figure 5A; *r* = 0.2639; *p* = 0.025) among different groups. Moreover, there was an obvious tendency for separating the bacterial profiles on genus level after VK2 supplementation (Figure 5B), and a great difference in the individual samples was found in the HS group, but little difference in the individual samples after VK2 supplementation (Figure 5B), which promulgated a positive action on genera after VK2 supplementation. Additionally, a total of 29 different bacteria after VK2 supplementation were screened among the three groups (Figure 5C; Supplementary Table 8).

### Verification of Functional Prediction Based on the Different Bacteria in High Salt-Induced Mice

**Figure 6:**
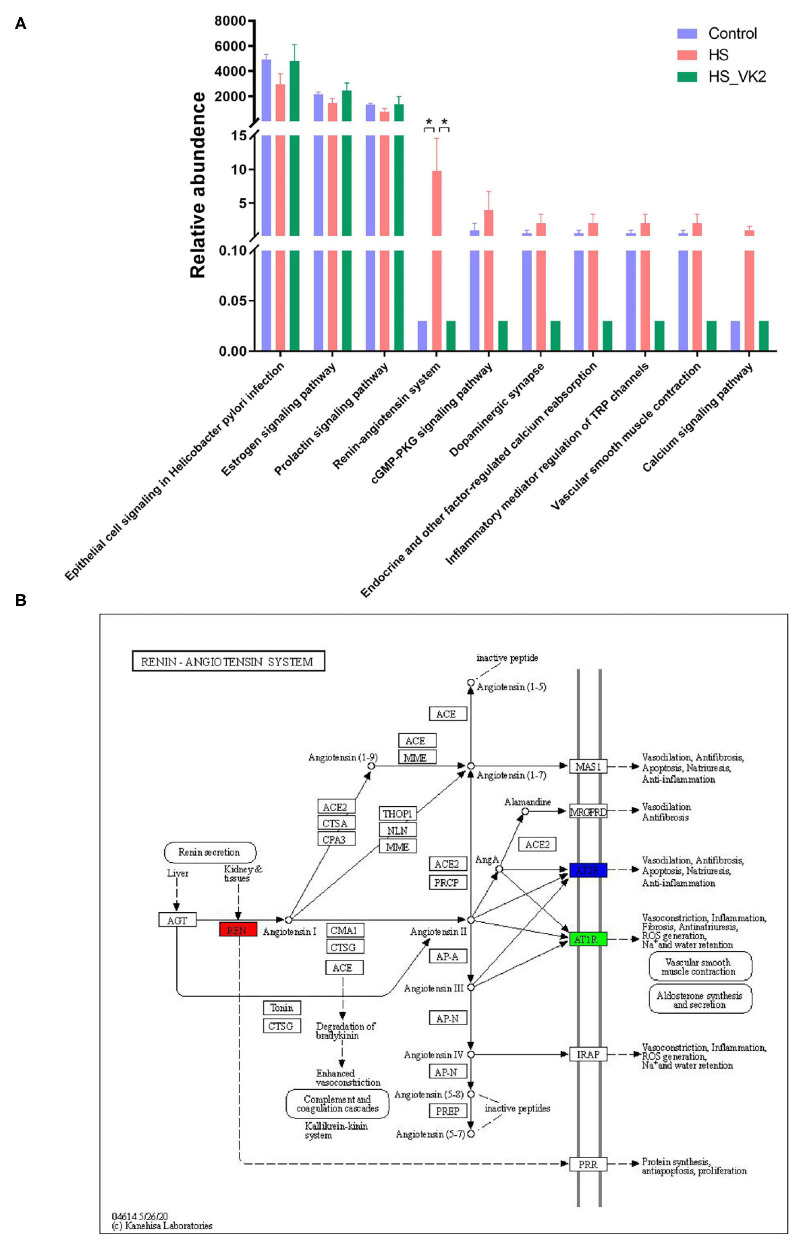
Verification of functional prediction based on the different bacteria. **(A)** Functional enrichment of PICRUSt2 based on the different bacteria. Data are presented as mean ± standard error of mean (SEM); *p < 0.05, *n* = 4; statistical comparisons were performed using Student *t*-test or one-way analysis of variance (ANOVA). **(B)** The mapper of Renin-angiotensin system.

To identify signaling pathways found through the previous network of VK2-treated salt-sensitive hypertension, functional analysis was performed using PICRUSt2 after VK2 supplementation. A total of 10 common signaling pathways were enriched and identified. As shown in Figure 5A, compared with the control group, decreased relative abundance was found in the signaling pathways in epithelial cell signaling in *Helicobacter pylori* infection, estrogen signaling pathway, and prolactin signaling pathway in the HS group; increased relative abundance was found in the renin–angiotensin system, cGMP-PKG signaling pathway, dopaminergic synapse, endocrine, and other factor-regulated calcium reabsorption, inflammatory mediator regulation of TRP channels, vascular smooth muscle contraction, and calcium signaling pathway in the HS group. Increased relative abundance was found in the signaling pathways in epithelial cell signaling in *Helicobacter pylori* infection, estrogen signaling pathway, and prolactin signaling pathway after VK2 supplementation; decreased relative abundance was found in the renin–angiotensin system, cGMP-PKG signaling pathway, dopaminergic synapse, endocrine, and other factor-regulated calcium reabsorption, inflammatory mediator regulation of TRP channels, vascular smooth muscle contraction, and calcium signaling pathway after VK2 supplementation (Figure 6A). Moreover, there was a greatest change multiple and statistical significance of the relative abundance in renin-angiotensin system (*p* < 0.05). Also, the mapper of the renin–angiotensin system is shown in Figure 6B combined with the findings in the previous network of VK2-treated salt-sensitive hypertension. All these results further revealed the fact that renin-angiotensin system was the potential mechanisms of VK2-treated salt-sensitive hypertension.

### Vitamin K_2_ Supplementation Inhibited Renin–Angiotensin System-Related Protein Expression in High Salt-Induced Mice

**Figure 7:**
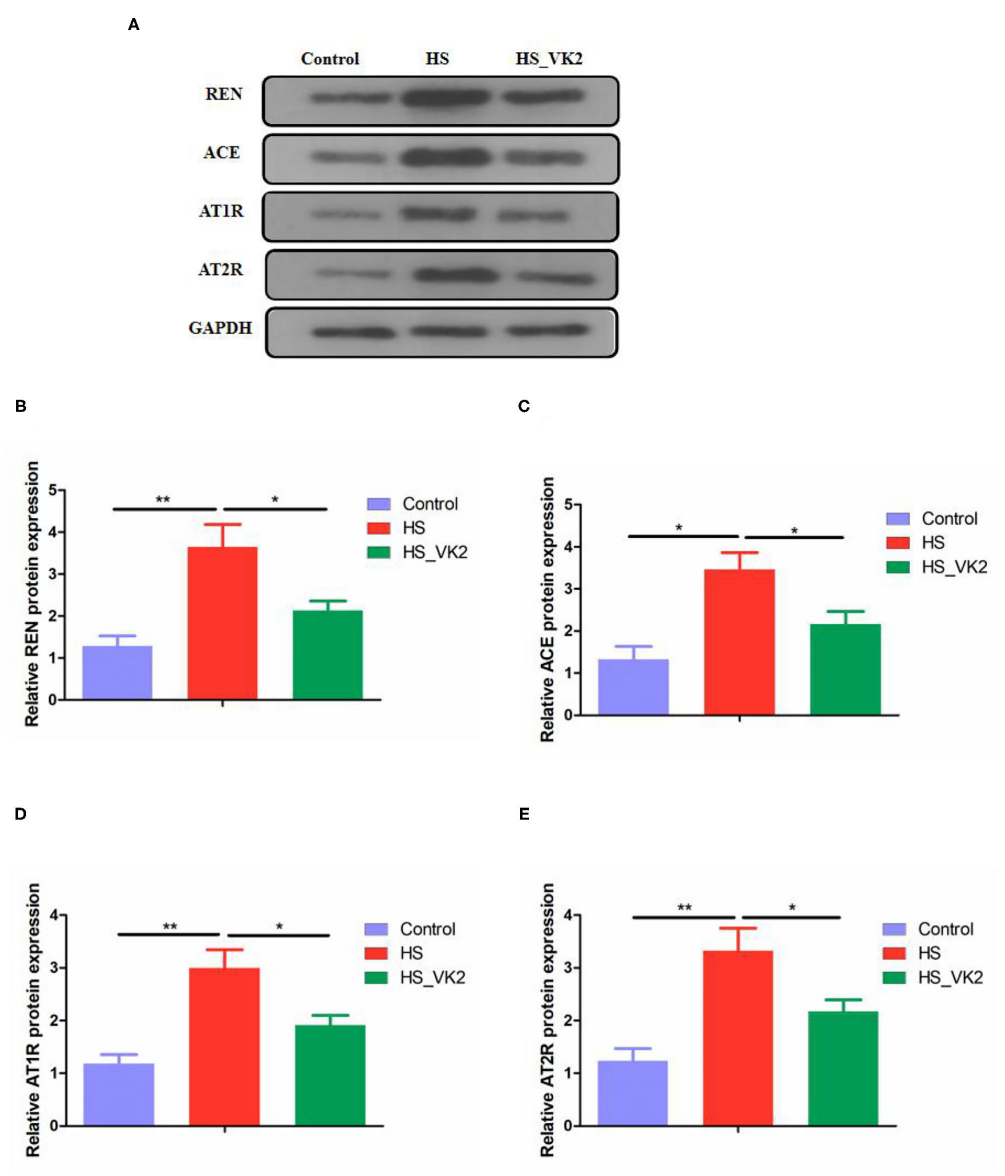
VK2 inhibited renin-angiotensin system-related proteins expression in high salt-induced mice **(A–E)**. Data are presented as mean ± standard error of mean (SEM); *p < 0.05 and **p < 0.01, *n* = 3; statistical comparisons were performed using Student *t*-test or one-way analysis of variance (ANOVA).

To further verify the effect of VK2 on the renin–angiotensin system, the renin–angiotensin system-related proteins expression (including REN, ACE, AT1R, and AT2R) in salt-induced mice were conducted. Significantly increased REN, ACE, AT1R, and AT2R proteins expression were found in the HS group compared with the control group. Moreover, significantly decreased REN, ACE, AT1R, and AT2R proteins expression were found after VK2 supplementation (Figures 7A–E). Obviously, the changes in the renin–angiotensin system-related proteins expression exhibited VK2 inhibited renin-angiotensin system-related proteins expression in salt-induced hypertensive mice, which was statistically significantly consistent with the previous biological and functional prediction analysis.

### Microbial Correlation

**Figure 8:**
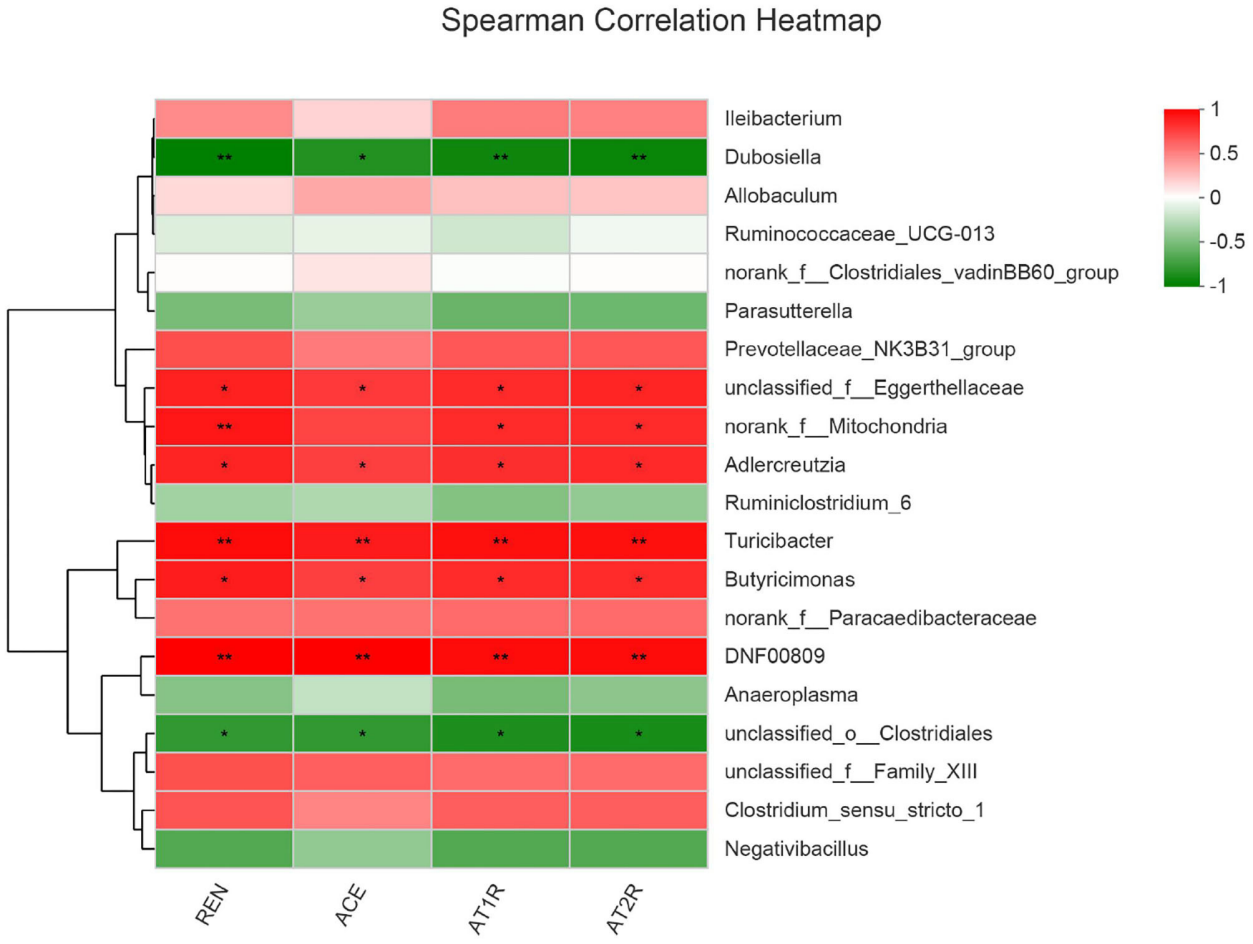
Microbial correlation with renin-angiotensin system-related proteins. Red represents positive correlation, whereas blue represents negative correlation; the darker the color, the stronger the correlation. 0.01 < *p ≤ 0.05, 0.001 < **p ≤ 0.01.

To reveal the relationship between differential bacteria and functional proteins in renin-angiotensin system-related proteins, Spearman correlation analysis of the renin–angiotensin system-related proteins and gut microbiota were performed. The results of spearman correlation analysis indicated that the 20 different bacteria such as *Dubosiella, Ileibacterium*, etc., had a positive or negative correlation with REN, ACE, AT1R, and AT2R in salt-induced mice (Figure 8).

## Discussion

Salt-sensitive hypertension is an important type of essential hypertension, which is a response to high salt intake. Modern studies have suggested that gut bacteria act as a participant and a target role for treatment in salt-sensitive hypertension (3, 4). Human studies have confirmed that VK2 is related to the prevention and treatment of cardiovascular diseases, and has revealed its curative effect on hypertension (9, 10, 19). However, these results lack more scientific evidence and molecular mechanisms. Therefore, this study focused on the molecular mechanisms of VK2 in the treatment of salt-sensitive hypertension, combined with network pharmacology and 16S rRNA sequencing strategy. In the current study, a total of 42 predictive targets of VK2-treated salt-sensitive hypertension were screened and identified accordingly. Moreover, the top 10 core genes of VK2 treated-salt-sensitive hypertension were screened as ALB, REN, IL10, SERPINE1, SRC, EGFR, F2, MAPK8, ESR1, and PLG. The network pharmacology-based findings from KEGG enrichment analysis revealed that VK2 treated salt-sensitive hypertension were mechanically associated with the neuroactive ligand-receptor interaction, complement and coagulation cascades, calcium signaling pathway, endocrine and other factor-regulated calcium reabsorption and renin-angiotensin system, etc. Additionally, a total of 29 different bacteria in animal experiment after VK2 supplementation were screened and functionally enriched using PICRUSt2. Ten common signaling pathways were identified in which the renin-angiotensin system was found to be the potential molecular mechanisms with the greatest change multiple and statistical significance. Finally, the renin-angiotensin system-related proteins expression exhibited that VK2 inhibited renin-angiotensin system in salt-induced mice, which was statistically, significantly consistent with the previous biological and functional prediction analysis.

On the basis of understanding the interaction network of “drug-target-disease,” the intervention and influence of drugs on the complex pathological network were observed through network analysis, and the interaction relationship between each node of the network was observed intuitively and clearly by using large-scale data integration, which provided a new platform for the mechanism target research of complex diseases (13, 14). The results of network pharmacology research showed that the core targets of VK2 in the treatment of salt-sensitive hypertension included as ALB, REN, IL10, SERPINE1, SRC, EGFR, F2, MAPK8, ESR1, and PLG. The findings of KEGG function enrichment showed that VK2-treated salt-sensitive hypertension was mechanically associated with the neuroactive ligand–receptor interaction, complement and coagulation cascades, calcium signaling pathway, endocrine, and other factor-regulated calcium reabsorption, renin–angiotensin system, etc. Therefore, the network pharmacology-based findings showed the potential molecular mechanisms of VK2-treated salt-sensitive hypertension.

Gut bacteria play an important role in the digestion and absorption of nutrients in food and are considered as therapeutic targets of many diseases and drugs (30, 31). It has become a hot research method in modern pharmacology to study the molecular mechanisms of drug treating diseases by focusing on gut bacteria (20, 21). In order to further verify the potential mechanisms of VK2 for salt-sensitive hypertension, we supplemented VK2 to intervene high-salt induced mice, and detected the characteristics of gut bacteria by 16S rRNA analysis. Some studies have shown that high-salt diet (8% NaCl) for 4 weeks can cause hypertension in mice (22, 23). The results of animal experiment showed that VK2 supplementation protected blood pressure and aortic vessels in salt-induced mice. Meanwhile, the results of the animal experiment showed VK2 supplementation reduced OTUs in gut bacteria of mice fed with a high-salt diet. The results revealed that there were differences in bacterial composition and structure after VK2 supplementation. Moreover, a total of 29 different bacteria were screened after VK2 supplementation including *Ileibacterium, Dubosiella, Allobaculum, Parasutterella, Ruminococcaceae_UCG-013*, etc. To identify signaling pathways found through the previous network of VK2-treated salt-sensitive hypertension, functional analysis was performed using PICRUSt2 after VK2 supplementation. The results showed that the signaling pathways of epithelial cell signaling in *Helicobacter pylori* infection, estrogen signaling pathway, and prolactin signaling pathway, renin–angiotensin system, cGMP-PKG signaling pathway, dopaminergic synapse, endocrine, and other factor-regulated calcium reabsorption, inflammatory mediator regulation of TRP channels, vascular smooth muscle contraction, and calcium signaling pathway were also found in the animal experiment after VK2 supplementation. Meanwhile, the greatest change multiple and statistical significance, which verified that the renin–angiotensin system was the potential mechanism of VK2 for salt-sensitive hypertension was found in the relative abundance of the renin–angiotensin system.

The renin–angiotensin system is considered as a peptidergic system with endocrine characteristics, with regard to the regulation of the blood pressure and hydro-electrolytic balance (24, 25). In the classical renin–angiotensin system, the renin cleaves its substrate angiotensinogen (Agt) forming the decapeptide angiotensin I (Ang I) that is in turn cleaved by the angiotensin-converting enzyme (ACE) to produce the angiotensin II (Ang II), which can affect the AT1R and AT2R, key players of this system (24, 25). In fact, it has been revealed that there are associations between insufficiency of fat-soluble vitamins and cardiovascular diseases (26). For example, long-term lack of vitamin D can lead to overactivation of the renin–angiotensin system, which is one of the mechanisms of blood pressure regulation (27, 28). In order to reveal the relationship between differential bacteria and functional proteins in the renin–angiotensin system-related proteins (including REN, ACE, AT1R, and AT2R), Spearman correlation analysis were conducted. The different bacteria, such as *Dubosiella, Ileibacterium*, etc., had a positive or negative correlation with REN, ACE, AT1R, and AT2R in salt-induced mice. It was showed that *Dubosiella* may have a role in inhibiting renin–angiotensin system including proteins (including REN, ACE, AT1R, and AT2R), whereas *Ileibacterium* may have a positive role. *Dubosiella newyorkensis* (belonging to genus *Dubosiella*), is related to many disease such as obesity, diabetes, abnormal lipid metabolism, etc., which was even used as a patented probiotic for many diseases (29). However, there are no other differential bacteria and diseases reported. The positive or negative correlation between the differential bacteria and functional proteins in renin–angiotensin system-related proteins (including REN, ACE, AT1R, and AT2R) reveal that the gut microbiota play an essential role in regulating blood pressure and the potential molecular mechanisms. These evidences show the relationship between differential bacteria and functional proteins, providing new research fields.

**Figure 9:**
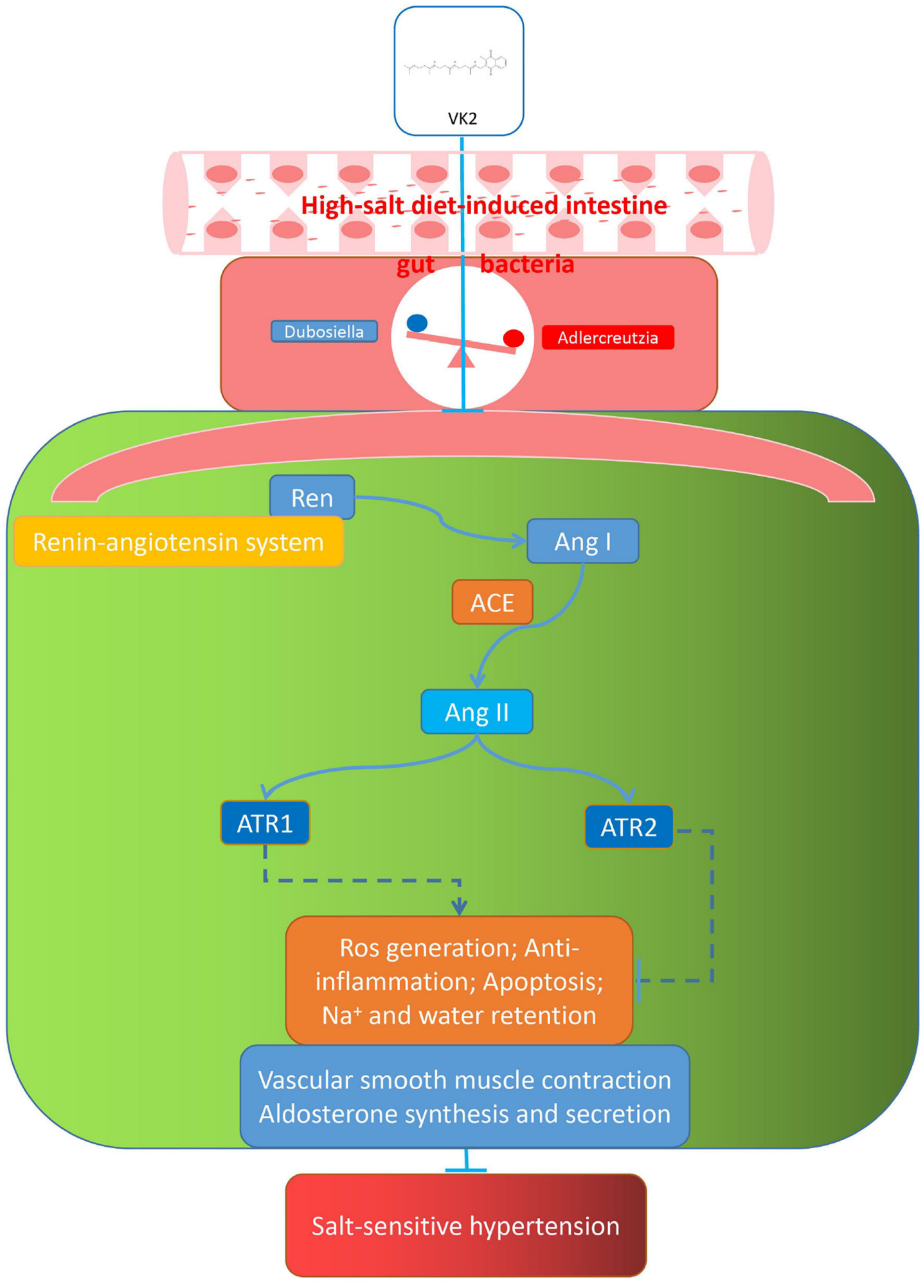
Representative cartoon with the possible mechanisms of VK2 for salt-sensitive hypertension.

The current study showed VK2-treated salt-sensitive hypertension by the specific inhibiting the renin–angiotensin system. Additionally, the renin–angiotensin system-related protein expression (including REN, ACE, AT1R, and AT2R) exhibited that VK2 inhibited the renin–angiotensin system-related protein expression in salt-induced hypertensive mice, which significantly verified the previous biological and functional prediction analysis. Accordingly, the representative cartoon with the potential mechanisms of VK2 for salt-sensitive hypertension is shown in Figure 9. However, such an integrated pharmacology and gut bacteria-based analysis further explored the potential mechanismic role of VK2 for salt-sensitive hypertension. More studies using clinical samples still need to be better investigated in the future. Obviously, the current study contributes to the development for a new preventive strategy of salt-sensitive hypertension as far as it is concerned.

## Conclusion

In the current bioinformatics and animal experiment verification used network and 16S rRNA sequencing-combined approach, the potential molecular mechanisms of VK2 related to salt-sensitive hypertension may be beneficially achieved by the specific inhibition of the renin–angiotensin system, contributing to provide the scientific evidence for the effective treatment of salt-sensitive hypertension.

## Data Availability Statement

The data presented in the study are deposited in the (NCBI SRA) repository, accession number (PRJNA690768).

## Ethics Statement

The animal study was reviewed and approved by the experimental animal ethics committee of Jinan University. Written informed consent was obtained from the owners for the participation of their animals in this study.

## Author Contributions

T-hL, M-hC, YX, and L-gC conceived and designed this study, analyzed the data, and wrote and revised the manuscript. W-qT, W-cT, ZJ, and Q-eL were responsible for the performance of animal experiments.

## Conflict of Interest

The authors declare that the research was conducted in the absence of any commercial or financial relationships that could be construed as a potential conflict of interest.

## Abbreviations

ANOVA: one-way analysis of variance
CHD: coronary heart disease
VK2: Vitamin K_2_
OTUs: operational taxonomic units.
